# Viral IRES Prediction System - a Web Server for Prediction of the IRES Secondary Structure *In Silico*

**DOI:** 10.1371/journal.pone.0079288

**Published:** 2013-11-05

**Authors:** Jun-Jie Hong, Tzong-Yuan Wu, Tsair-Yuan Chang, Chung-Yung Chen

**Affiliations:** 1 Department of Bioscience Technology, Chung Yuan Christian University, Chung-Li, Taiwan; 2 Center for Nanotechnology and Institute of Biomedical Technology, Chung Yuan Christian University, Chung-Li, Taiwan; 3 R&D Center of Membrane Technology, Chung Yuan Christian University, Chung-Li, Taiwan; 4 Information Management Department, Ming Chuan University, Guishan Township, Taoyuan, County, Taiwan; University of Rome, Italy

## Abstract

The internal ribosomal entry site (IRES) functions as cap-independent translation initiation sites in eukaryotic cells. IRES elements have been applied as useful tools for bi-cistronic expression vectors. Current RNA structure prediction programs are unable to predict precisely the potential IRES element. We have designed a viral IRES prediction system (VIPS) to perform the IRES secondary structure prediction. In order to obtain better results for the IRES prediction, the VIPS can evaluate and predict for all four different groups of IRESs with a higher accuracy. RNA secondary structure prediction, comparison, and pseudoknot prediction programs were implemented to form the three-stage procedure for the VIPS. The backbone of VIPS includes: the RNAL fold program, aimed to predict local RNA secondary structures by minimum free energy method; the RNA Align program, intended to compare predicted structures; and pknotsRG program, used to calculate the pseudoknot structure. VIPS was evaluated by using UTR database, IRES database and Virus database, and the accuracy rate of VIPS was assessed as 98.53%, 90.80%, 82.36% and 80.41% for IRES groups 1, 2, 3, and 4, respectively. This advance useful search approach for IRES structures will facilitate IRES related studies. The VIPS on-line website service is available at http://140.135.61.250/vips/.

## Introduction

Translation initiation can be described as a scanning model triggered by a cap- and 5’ end-dependent mechanism, or can be mediated by a cap- and 5’ end-independent manner through an RNA element termed as “internal ribosomal entry site” (IRES). The scanning machine recognizes and binds to the methylated 5’-end cap structure of a mRNA and scans linearly downstream until it reaches an AUG codon for the initiation of protein translation [1]. In contrast to the canonical translation initiation, the IRES directs the ribosomal translation due to form specific secondary and tertiary structures that interact directly with the translational machinery. IRES elements were first described in the 5' nontranslated region of mRNAs of the *Picornaviridae*, which lacks a methylated cap structure at the 5’ end [2]. The IRES may have an important role as a virulence factor, in addition, the identification of IRES element of pathogenic viruses is also a key point for the treatment of the viruses-infected diseases. Moreover, the IRES element can be applied in the development of bi-cistronic expression vector, an important tool for the biotechnology. Thus, it is important to develop a bioinformatic tool for the prediction and identification of IRES element(s) in a virus’s genome.

According to RNA structures, IRESs are functionally classified into four major structural groups: Group 1 (ie., Cricket paralysis virus; CrPV) [3], Group 2 (ie., Hepatitis C virus; HCV) [4], Group 3 (ie., Encephalomyocarditis virus, EMCV) [5] and Group 4 (ie., Poliovirus; PV) [1,6]. The IRES element prediction might depend on RNA structure similarity because of the functional contraction. The ameliorative RNA structure prediction will therefore be useful to enhance the accuracy of secondary structure prediction of IRES elements. We have developed an IRES search system named IRSS that combined two RNA structure prediction models: comparative sequence analysis, and minimum free energy structure [7]. Comparative sequence analysis has a 97% accuracy of base pairs in ribosomal RNA secondary structures, and minimum free energy (MFE) structure prediction can predict the structure of a single RNA sequence with an average of 73% accuracy [8]. However, comparative sequence analysis is not useful to predict the mRNA regulatory motifs such as IRES [9,10].

Recently, RNA pseudoknot structure has been demonstrated to play important roles in many biological processes, including building of the catalytic core of some ribozymes [11]. From cryo-electron microscopy structure information of HCV IRES, the pseudoknot element might bind to the initiation codon of the mRNA that has attached the binding cleft with the 40S ribosomal subunit [12,13]. The intergenic region (IGR) IRES of *Plautia stali* intestine virus contains three pseudoknot structures; two located on 5′-terminal 143 nucleotides for binding of the IGR IRES to the 40S ribosome, and one 3′-terminal pseudoknot involved in decoding of the non-AUG codon used for initiation [14]. Thus, the pseudoknot structure might be one of important parameters in determining the IRES elements and might be used to improve the accuracy of IRES prediction. The program, pknotsRG, adopted an algorithm to calculate the thermodynamic stability of pseudoknots, which can predict a restricted class of pseudoknots [15].

For the RNA structure and sequence comparative tools, many pattern searching programs and web services have been developed, such as Rfam from the Sanger Institute [16]. Rfam adopted multiple RNA sequence alignments using covariance models to represent consensus primary sequences of non-coding RNA families. Moreover, there are twelve IRES models built upon consensus sequences in Rfam database. Unfortunately, the lower homology between different IRES groups will cause inaccuracy of prediction using primary sequences [9,10]. The RNA structure prediction will therefore be useful to enhance the accuracy of de novo secondary structure prediction of IRES elements. To develop a new IRES search tool which is able to predict all four viral IRES groups, the viral IRES prediction system (VIPS) was constructed and based on secondary structure prediction, structure comparison and pseudoknot structure calculation. In contrast to Rfam, IRSS, the previous prediction system and VIPS will be more specific for IRES prediction [7]. VIPS will scan neighboring regions for structure prediction and avoid short consensus primary sequence problems to improve IRES structure predictions. The VIPS also added pknotsRG that will enhance the accuracy of predicting the IRES structures with regards to the function of pseudoknot binding with 40S ribosome. Previous IRES search system (IRSS) can provide up to 72.3% accuracy of secondary structure prediction for IRES group 2 [7]. The VIPS has higher accuracy than IRSS and is a useful search platform for IRES prediction due to more competent standard IRES elements and parameters of VIPS. The web searching service of VIPS provides a new IRES search tool which can assist in defining the IRES elements. In addition, the VIPS will also provide a useful source for IRES location before experimental study. The VIPS will be a public resource, and can facilitate the scientific community not only to as an analyzing tool, but also as means of communication by providing feedbacks.

## Materials and Methods

**Figure 1:**
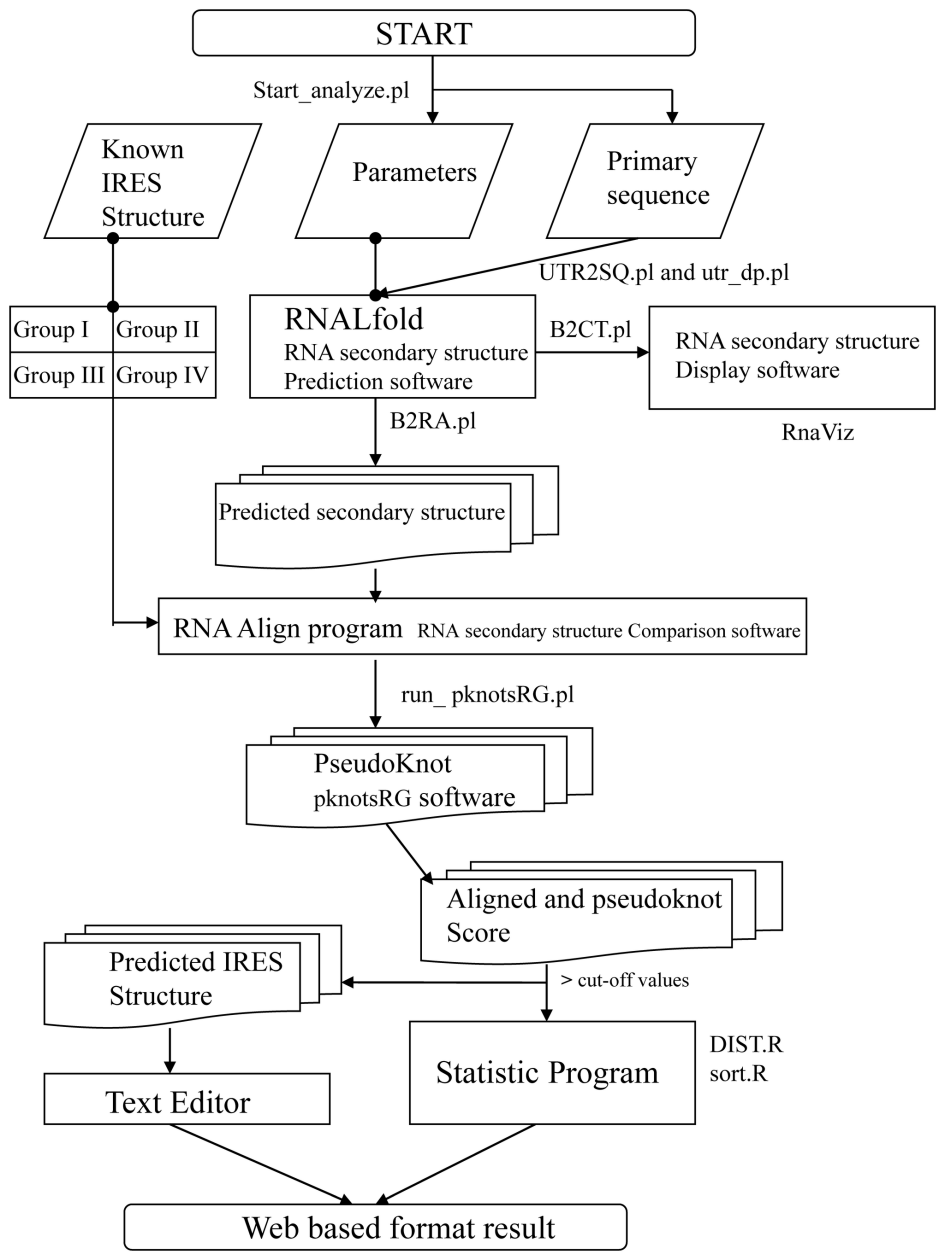
Flowchart of VIPS. The FASTA/plain text RNA primary sequence ran by RNAL fold and compared with known IRES structure by RNA Align and pknotsRG programs. The IRES structures are displayed by RnaViz software and alignment results can be edited by any text editor. The eight private programs are pointed beside arrow symbols. The sort codes are shown in Supplementary file (Methods S1).

Three key steps are the backbone of the viral IRES prediction system (VIPS): 1) RNA folding, 2) RNA secondary structure comparison and 3) pknotsRG program. First, RNAL fold program functions to predict the RNA secondary structure using the minimum free energy method [17]. Next, the RNA secondary structure comparison matches the known IRES structures executed by RNA Align program [18]. Finally, the pknotsRG calculates the pseudoknot score from potential IRES structures [15]. In our designed VIPS, the primary RNA sequence input in the search flowchart (see Figure 1), with default length parameter (L=250, previous results [7]), is transferred as a raw RNA sequences into RNAL fold input format by perl scripts (UTR2SQ.pl and utr_dp.pl) (Methods S1) [7]. The Start_analyze.pl is the major control batch program to link each stage of VIPS. In RNA align software, two factors are considered to evaluate the IRES elements that can be predicted by our VIPS, distance score (DIST) and alignment match length (ALEN). DIST represents the score of secondary structure in comparison with the default score of each RNA structure (base-deletion, base-mismatch, arc-mismatch, are-removing, arc-altering and arc-breaking) adopted in RNA align software. Because DIST value will increase concomitantly with longer alignment length, DIST score fails to specify the significance of matched structures from shorter and bigger alignment sequences. Therefore, DIST and ALEN are transformed into a ratio which is defined as R= ALEN/DIST [7]. The R values are collected from all predicted IRES elements including known IRES and potential candidate IRES elements. Linear discriminant analysis (LDA) analyzes all R values to make a discriminant line that distinguishes candidate IRES group and non-IRES group. The error rate of VIPS is estimated in comparison of known IRES structures with candidate IRES elements. All parameters were succeeded from our previous IRSS setting [7]. The output data of RNAL fold program is re-transformed into RNA Align format by B2RA.pl program (Methods S1). For RNA view, B2CT.pl (Methods S1) changes the predicted RNA secondary structure into “connect file format” (*.ct) which will read by RnaViz [19] to display in screen and print. Two output files, Aligned structure and Alignment score files, were generated by RNA Align software. 2 statistical programs, DIST.R and sort.R, were applied to select all predicted RNA structures with R scores higher than best cut-off value [7]. The perl script, run_pknotsRG.pl, re-formats all candidate RNA structure into input format of pknotsRG software (Methods S1). All of the output results of RNA Align and pknotsRG software were evaluated their value by statistic programs. The predicted figure of RNAL fold program and text results of RNA Align and pknotsRG software were showed as web page while their values are higher than cut-off value.

The VIPS has been implanted with known IRES elements as standard structures. For example, twelve IRES models were built upon the consensus sequences in Rfam database. (http://www.sanger.ac.uk/Software/Rfam/). Thus, these IRES consensus secondary sequences are the major templates for RNA fold program, a part of VIPS. In VIPS, if the RNAL fold program predicted an IRES element that cannot match any IRES models of Rfam or fetch at least two homolog IRESs from related species, the input data will be discarded.

To evaluate the precision of VIPS, known IRES elements, such as in the IRES database (http://www.iresite.org), and the IRES elements of HCV domain III (accession number: AF177037), poliovirus (accession number: V01149), encephalomyocarditis virus (EMCV; accession number: X87335), and cricket paralysis virus (accession number: AF218039), were input in the VIPS as training data. Also, the entire UTR database (UTRdb, http://www.ba.itb.cnr.it/UTR/) and a part of viral database (http://www.ncbi.nlm.nih.gov) sequences were input into the VIPS to estimate the accuracy of IRES prediction. The distribution of pseudoknot value of pknotsRG plus the R value of VIPS were analyzed to make a discriminant line that distinguishes candidate IRES group and non-IRES group for each IRES type. The experimental IRES elements of IRES database were applied to compare with the results of UTR database searched by VIPS. The error rate of VIPS was therefore calculated to assess the accuracy of VIPS. Finally, randomly selected 500 virus genome data from NCBI were applied to test VIPS to predict IRES elements of whole viral genomes (Data not showed).

The VIPS web service has been built in Linux platform in IBM server X3400. The automatic batch system will execute the customers’ requests and run through all programs (Figure 1) to compare four individual IRES type plus pseudoknot parameters and create a plain text file will be sent back through email to the user due to long CPU running time.

##  Results

### Evaluation of VIPS by four individual IRES groups

**Figure 2:**
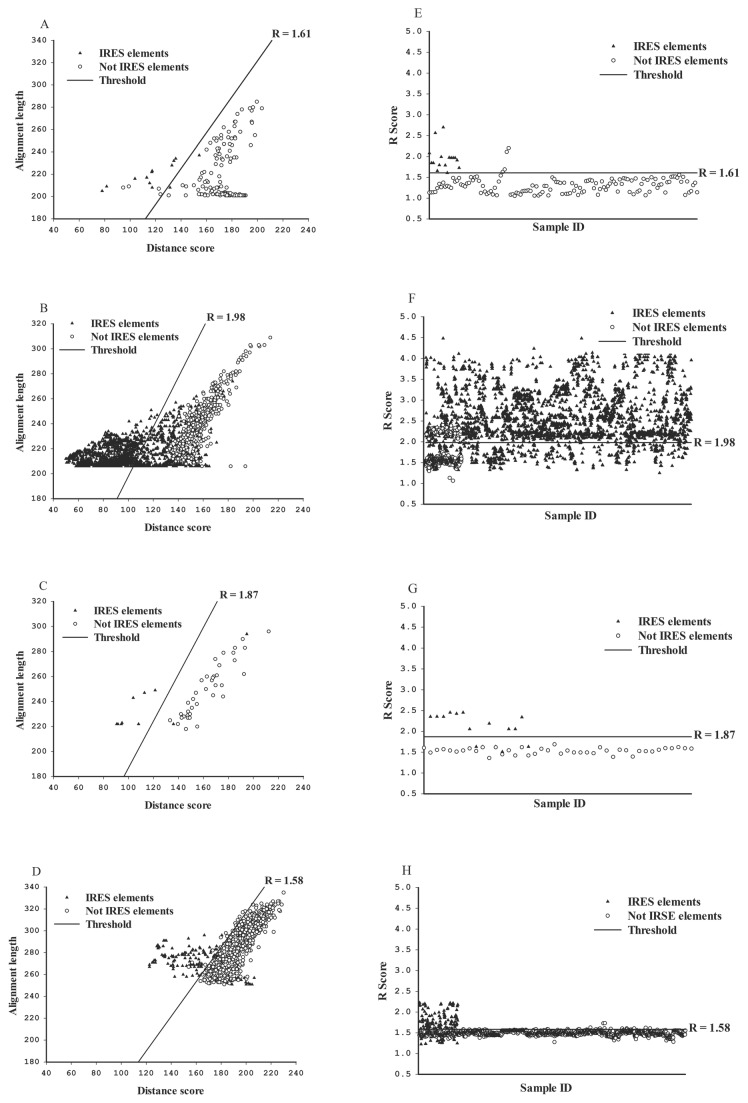
The distribution of R and pseudoknot parameters from positive and negative group of four IRES groups by VIPS analysis. The distribution of four IRES groups calculated by R and pseudoknot parameters in positive group and negative group. The R scores and pseudoknot scores were demonstrated according to IRES group 1 (a and e), IRES group 2 (b and f), IRES group 3 (c and g) and IRES group 4 (d and h). Linear discriminant analysis was applied to determine the cut-off line of the R score in each group shown as (a), (b), (c) and (d). Distribution of positive and negative IRES elements that were calculated and summarized from R and pseudoknot values are shown as (e), (f), (g) and (h).

In order to develop a new IRES prediction system based on the previous IRES element search system (IRSS)[7], different standard templates and training data were inputted into VIPS which is ran by RNAL fold and RNA Align programs with length parameter (L = 250, default). The standard structures were fetched from four known groups of IRES elements based on Cricket paralysis virus (Group 1, accession number: AF218039), Hepatitis C virus (Group 2, accession number: AF177037), Encephalomyocarditis virus (Group 3, accession number: X74312.1) and Poliovirus (Group 4, accession number: V01148.1). Those standard IRES templates were applied into VIPS to calculate the appropriate individual R value and pseudoknot value from RNAL fold, RNA Align and pknotsRG programs. The R value of VIPS presents a score for match length (ALEN) divided distance score (DIST) that distributes into two separate groups, IRES-candidate group and negative group, when the cut-off value was determined [7]. For positive groups, all verified IRES elements (Table S1) of the four viral families (groups 1~4) fetched from NCBI GenBank (http://www.ncbi.nlm.nih.gov) and Rfam database (http://www.sanger.ac.uk/Software/Rfam/) were run through VIPS to calculate and classify into four IRES groups. Their R and pseudoknot values were collected as training data. For negative groups, the all known coding sequences without IRES elements of Poliovirus, Encephalomyocarditis virus, Hepatitis C virus and Cricket paralysis virus were input into VIPS to analyze their R and pseudoknot values. For each IRES group, the cut-off values were estimated from the positive group and negative group by linear discriminant analysis. The cut-off value is 1.61, 1.98, 1.87, and 1.58 of R value for IRES group 1, 2, 3, 4 respectively (Table 1; Figure 2a, 2b, 2c and 2d). The sensitivity and specificity of each IRES group are shown in Table 1. 

**Table 1:** The accuracy of the predicted IRES elements for IRES group 1, 2, 3 and 4 by VIPS.

	**IRES group**	**1**	**2**	**3**	**4**
**R score**	Cut-off value	1.61	1.98	1.87	1.58
	Average R score of positive group	1.90±0.29	2.42±0.62	2.05±0.34	1.68±0.25
	Average R score of negative group	1.29±0.18	1.53±0.07	1.53±0.07	1.49±0.05
	*P*	<0.001	<0.001	<0.001	<0.001
	Sensitivity	87.50%	80.52%	64.71%	56.34%
	Specificity	97.06%	100%	100%	98.85%
	Accuracy rate	92.28%	90.26%	82.36%	77.60%
**pseudoknot prediction**	Positive group contains pseudoknot structure	81.25%	15.70%	11.76%	40.85%
	Negative group contains pseudoknot structure	16.18%	14.70%	9.52%	35.94%
**R score plus pseudoknot prediction**	Sensitivity	100%	81.59%	64.71%	62.44%
	Specificity	97.06%	100%	100%	98.37%
	Accuracy rate	98.53%	90.8%	82.36%	80.41%

All values are expressed as means ±SEM. *P* values were calculated by *t*-test.

In IRES group 4, the average R score of positive group is 1.68 ± 0.25 (mean ± SD) and of negative group was 1.49 ± 0.05 (*P*<0.001, Table 1). Thus, after linear discriminant analysis, false negative was 43.66% and false positive was 1.15% for IRES group 4, wherein the cut-off value is 1.58. For IRES group 3, VIPS showed higher accuracy to predict this type than group 4. The average R-score of IRES group 3 for both positive and negative groups were 2.05 ± 0.34 and 1.53 ± 0.07 (*P*<0.001), respectively. Therefore, the false negative and positive were estimated as 35.29% and 0.00% for IRES group 3, respectively, if cut-off value is 1.87. For IRES group 2, VIPS showed 19.48% false negative and 0.00% false positive in 1.98 cut-off value determined by linear discriminant analysis between positive (2.42 ± 0.62) and negative (1.53 ± 0.07) groups (*P*<0.001). For IRES group 1, VIPS represented 12.50% for false negative and 2.94% for false positive in 1.61 of cut-off value which analyzed from positive (1.90 ± 0.29) and negative (1.29 ± 0.18) groups (*P*<0.001). The accuracy rate of VIPS for group 1, 2, 3 and 4 were 92.28%, 90.26%, 82.36% and 77.60%, respectively (Table 1).

The pseudoknot structure might enhance the prediction ability for IRES elements. The 40.85% of the positive group and 35.94% of the negative group contained predicted pseudoknot structures from IRES group 4 (Table 1, Figure 2h). For IRES group 3, 11.76% of the positive group and 9.52% of the negative group have been predicted to form pseudoknot structures (Table 1, Figure 2g). For IRES group 1 and 2, potential pseudoknot structures appeared in 81.25% and 15.70% of the positive groups, respectively. In contrast to negative groups, 16.18% and 14.70% of IRES group 1 and 2 contained candidate pseudoknot structures (Table 1, Figure 2e and 2f). The combination of R values and pseudoknot prediction increased the accuracy from 92.28% to 98.53% in group 1 and 90.26% to 90.80% in group 2 of VIPS prediction (Table 1). Moreover, the pseudoknot calculation was able to enhance the precision of VIPS system up to 80.41% in IRES group 4, but not in IRES group 3 (Table 1).

To validate the specificity of VIPS, the standard IRES elements were examined and compared with different IRES groups by VIPS (Table S2). Each standard IRES element showed specificity in higher R score to distinguish between the specific IRES group and other three IRES groups. Moreover, while the standard IRES group 2, and 3 compared to different IRES groups under VIPS estimation, no any false positive results occurred. However, groups 2, 3 and 4 of IRES element or non-IRES sequences were compared with Cripavirus IRES (group 1 standard) and showed a R-score range of 1.44 ~1.53, which is lower than the standard R-score (1.90±0.29) of IRES group 1 (Table S2) but has 0.24% and 2.11% of false positive in group 2 and 4 negative controls individually. For group 4 standard, PV IRES, has 0.13% and 1.69% of false positive in groups 2 IRES element and negative control respectively, in comparison by VIPS study (Table S2).

In order to evaluate the accuracy rate of the known IRES elements, the IRES information in Rfam database (http://www.sanger.ac.uk/Software/Rfam/) (excluding the four IRES standard elements) were analyzed in VIPS. From the verified IRES data of Rfam database, there were 16, 3096, 17 and 213 records for IRES group 1, 2, 3, and 4, respectively (Table 2). VIPS showed 100% (16/16, IRES group 1), 81.59% (2526/3096, IRES group 2), 64.71% (11/17, IRES group 3) and 62.44% (133/213, IRES group 4) prediction rates with pseudoknot function. From Table 2 and Rfam IRES search, the VIPS has inferior prediction rates without pseudoknot function and showed 87.50% (14/16, IRES group 1), 78.78% (2439/3096, IRES group 2), and 56.34% (120/213, IRES group 4) prediction rates.

**Table 2:** Statistical results of the predicted IRES elements from Rfam, UTR and virus databases by VIPS.

	**pseudoknot function**	**IRES group 1**	**IRES group 2**	**IRES group 3**	**IRES group 4**
**IRES data (Rfam, n=3342)**	**+**	1.96±0.28 (n=16/16)	2.64±0.53 (n=2526/3096)	2.28±0.15 (n=11/17)	1.82±0.20 (n=133/213)
**Prediction rate**		100%	81.59%	64.47%	62.44%
**IRES data (Rfam, n=3342)**	**-**	1.95±0.28 (n=14/16)	2.61±0.54 (n=2439/3096)	2.28±0.15 (n=11/17)	1.84±0.20 (n=120/213)
**Prediction rate**		87.50%	78.78%	64.47%	56.34%
**UTR database (n=42768)**	**+**	1.64±0.03 (n=2622)	2.00±0.02 (n=44)	0.00±0.00 (n=0)	1.61±0.02 (n=220)
**UTR database (n=42768)**	**-**	1.63±0.02 (n=542)	0.00±0.00 (n=0)	0.00±0.00 (n=0)	1.60±0.02 (n=145)
**Virus database (n=447861)**	**+**	1.64±0.06 (n=743)	2.51±0.57 (n=37)	2.10±0.12 (n=19)	1.62±0.05 (n=172)
**Virus database (n=447861)**	**-**	1.66±0.11 (n=201)	2.69±0.60 (n=26)	2.10±0.12 (n=19)	1.62±0.06 (n=145)

All values are expressed as means±SEM.

### Evaluation of VIPS by UTR Database Scanning

To estimate the prediction of human cellular IRES elements by VIPS, the human 5’UTR information from UTR database (42768 records in total without redundant sequences) was scanned to predict IRES elements and compared with a known IRES database which has experimentally verified IRES elements (http://rfam.sanger.ac.uk/ and http://www.iresite.org). 687 records (1.61%) were predicted as potential IRES elements from VIPS without pseudoknot function. With pseudoknot function, 6.65% ((2622+220)/42768) of human 5’UTR records were predicted as IRES candidates. The top 15 predictions (R value over 1.70) of VIPS scanned human 5’UTR are shown in Table 3. However, VIPS can fetch 21.98% of the experimentally verified human cellular IRES elements from UTR database (data not showed). The outcome the UTR database scanning proved that the VIPS is able to predict cellular IRES elements but is inferior than viral IRES prediction.

**Table 3:** Top 15 records of VIPS predicted potential IRES elements from human 5’UTR of UTR database.

L*	**Accession No**	**Position**	**SLEN^*§*^**	**R and pseudoknot prediction**	**Description**
165	NM_152377	1-165	165	1.76|N	5'UTR in Homo sapiens chromosome 1 open reading frame 87 (C1orf87), mRNA.
337	NM_001080551	169-337	169	1.73|N	5'UTR in Homo sapiens chromosome 9 open reading frame 84 (C9orf84), transcript variant 2, mRNA.
265	NM_002803	75-265	191	1.73|Y	5'UTR in Homo sapiens proteasome (prosome, macropain) 26S subunit, ATPase, 2 (PSMC2), mRNA.
288	NM_015239	127-285	159	1.72|Y	5'UTR in Homo sapiens ATP/GTP binding protein 1 (AGTPBP1), mRNA.
470	NM_030571	251-465	215	1.72|Y	5'UTR in Homo sapiens Nedd4 family interacting protein 1 (NDFIP1), mRNA.
205	NM_001135811	28-199	172	1.72|Y	5'UTR in Homo sapiens family with sequence similarity 60, member A (FAM60A), transcript variant 1, mRNA.
359	NM_015454	165-359	195	1.71|Y	5'UTR in Homo sapiens La ribonucleoprotein domain family, member 7 (LARP7), transcript variant 2, mRNA.
282	NM_001007022	107-278	172	1.71|N	5'UTR in Homo sapiens outer dense fiber of sperm tails 2-like (ODF2L), transcript variant 2, mRNA.
177	NM_001003790	1-169	169	1.71|Y	5'UTR in Homo sapiens ER lipid raft associated 2 (ERLIN2), transcript variant 2, mRNA.
338	NM_001076785	171-337	167	1.70|N	5'UTR in Homo sapiens solute carrier family 7 (cationic amino acid transporter, y+ system), member 6 (SLC7A6), transcript variant 2, mRNA.
215	NM_001003927	10-211	202	1.70|N	5'UTR in Homo sapiens ecotropic viral integration site 2A (EVI2A), transcript variant 1, mRNA.
977	NM_032779	808-976	169	1.70|N	5'UTR in Homo sapiens coiled-coil domain containing 142 (CCDC142), mRNA.
442	NM_024650	217-436	220	1.70|Y	5'UTR in Homo sapiens chromosome 11 open reading frame 80 (C11orf80), mRNA.
537	NM_024650	312-531	220	1.70|Y	5'UTR in Homo sapiens chromosome 11 open reading frame 80 (C11orf80), mRNA.
686	NM_002270	500-682	183	1.70|N	5'UTR in Homo sapiens transportin 1 (TNPO1), transcript variant 1, mRNA.

* length of sequence fragments inputted into VIPS.

**^§^** predicted IRES sequence length.

VIPS showed 1.27% (542/42768), 0.00%, 0.00% and 0.33% (145/42768) of predicted IRES group 1, 2, 3 and 4, respectively from the human 5’UTR of UTR database analysis (w/o pseudoknot, Table 2). To confirm these candidate cellular IRES elements by the experimentally verified cellular IRES elements(http://www.iresite.org), 21.06% and 25.53% (without pseudoknot) of VIPS predicted IRES elements group 1 and 4 were verified (data not showed). Moreover, the major group is the Zinc finger genes from those IRES group 1 and 4 candidates (R value between 1.59 and 1.70, Table S3).

### Evaluation of VIPS by virus database scanning

To examine the prediction ability of IRES elements for viral genomes by VIPS, the sequence information of the four genera, Cripavirus, Hepacivirus, Cardiovirus and Enterovirus, and randomly selected 500 viral genomes without redundancy sequences (447861 records in total that are included 330728 records from 500 viral genomes but excluded the viral sequences of four standard IRES groups used in VIPS, data not showed) were fetched to predict IRES elements and also compared with a known viral IRES elements (http://www.iresite.org). However, the known viral IRES elements of Rfam data were also excluded. The 971 records (971/447861 = 0.22%, Table 2) were predicted as potential IRES elements from VIPS with pseudoknot function and the top 15 data of IRES prediction are shown in Table 4. The Drosophila melanogaster gypsy transposable element, *Plautia stali* intestine virus, Cricket paralysis virus, *Ectropis obliqua* picorna-like virus might belong to IRES group 1. In addition, Hepatitis GB virus B was predicted as an IRES group 2 structures. For IRES group 3, Foot-and-mouth disease virus, *Equine rhinitis* A virus, Theiler's murine encephalomyelitis virus were predicted. Moreover, the Human coxsackievirus, Human enterovirus, Poliovirus, Human rhinovirus were considered as IRES group 4. 

**Table 4:** Top 15 records of VIPS predicted potential IRES elements from viral IRES database.

L*	**Accession No**	**Position**	**SLEN^*§*^**	**R and pseudoknot prediction**	**Description**
416	AJ277947	241-427	187	2.40|N	Hepatitis GB virus B genomic RNA.
383	M67463	140-363	224	2.35|Y	Hepatitis C virus subtype 1a, strain H, complete genome.
1040	M16020	678-906	229	2.34|N	Theiler's murine encephalomyelitis virus (TMEV) RNA polyprotein, complete genome.
192	NC_003924	6047-6214	168	2.20|N	Cricket paralysis virus (Dicistroviridae) nonstructural polyprotein and structural polyprotein genes, complete genome.
363	NC_001461	173-404	232	2.17|Y	Bovine viral diarrhea virus 1, complete genome.
461	AJ133357	661-875	215	2.16|N	Foot-and-mouth disease virus (FMDV) strain C, isolate c-s8c1, genomic RNA.
712	L43052	430-651	222	2.00|N	Equine rhinitis A virus, genome incomplete at the 5'-end.
748	DQ060149	456-655	200	1.80|N	Human enterovirus 71 strain pinf7-54A from Taiwan, complete genome.
604	X02316	425-613	189	1.74|N	Human rhinovirus (HRV) 5'-UTR.
145	NC_003779	6004-6145	142	1.74|N	Plautia stali intestine virus (Dicistroviridae), complete genome.
742	K01392	404-640	237	1.73|N	Poliovirus P3/Leon/37 (type 3), complete genome.
390	NC_005092	160-380	221	1.70|Y	Ectropis obliqua picorna-like virus, complete genome.
750	AY752946	438-650	213	1.67|N	Human coxsackievirus B3 strain 20, complete genome.
261	AF033821	635-781	147	1.63|N	Drosophila melanogaster gypsy LTR-transposable element, full-lenght RNA.
330	AF033821	322-516	195	1.62|N	Drosophila melanogaster gypsy LTR-transposable element, full-lenght RNA.

* length of sequence fragments inputted into VIPS.

**^§^** predicted IRES sequence length.

To analyze each group, five top candidate virus families, Bat coronavirus, *Honeysuckle ringspot* virus, Tomato leaf deformation virus, *Euprosterna elaeasa* virus, *Lactococcus* phage, were found as potential IRES group 4 by VIPS searches (Table S4). For IRES group1, five candidate virus families were *Lactococcus* phage, Watermelon silver mottle virus, Human parainfluenza virus, *Hyposoter fugitivus* ichnovirus, *Acidianus* rod-shaped virus. These results demonstrated that VIPS can predict IRES elements from virus database and viral genomes.

### Web-based tools of VIPS

The VIPS tool is available as a web-based on-line search at http://140.135.61.250/vips/. All of the original RNA prediction software, perl-script programs and batch files have been implanted into a Web server and executed automatically. The input sequences are in plain text format limited with less than 5000 nucleotides. After VIPS prediction, all of the results with R score that are higher than cut-off values in individual IRES groups plus pseudoknot prediction will be shown as output. Those data include potential IRES sequences, predicted secondary structures, R score, pseudoknot prediction and their minimum free energies values for each structure. The results are showed in plain text format of web-page and will be sent through e-mail that can be read by any word processing software. In web-based VIPS, the default L parameter is 250, the cutting R values are 1.61, 1.98, 1.87, and 1.58 for IRES group 1, 2, 3, and 4, respectively. The users are able to adjust the cutting R values to modify the search criterion. In addition, the pseudoknot parameter can be set on/off for individual calculation to enhance the prediction of VIPS. The VIPS web tool is ran in a Linux workstation with Ubuntu 10.10 operation system.

## Discussion

IRES elements have been applied as gene expression tools. The functions and structures of IRESs have been studied by functional and mutational assay on different IRES elements. The development of the IRES element prediction system will help scientists predict the potential IRES elements prior to experimentations. However, most of the current software aims to predict the RNA secondary structure but not specifically predict the IRES elements, an example as Mfold [20]. To verify the accuracy of VIPS, IRES elements from three major related databases; experimentally verified IRES database (http://www.iresite.org), Rfam database (http://rfam.sanger.ac.uk/), and UTR database (http://www.ba.itb.cnr.it/UTR/) were collected and applied in our study. This helped in building a better and more useful IRES search system than the previous version, IRSS, which has been operated for over 2 years. The sensitivity of IRSS is less than 72% in IRES group 2 (IRES type 3), moreover, other IRES groups showed 40~70% accuracy in IRSS. The VIPS showed 92.28%, 90.26%, 82.36%, and 77.60% of accuracy rate for IRES group 1, 2, 3 and 4, respectively, without pseudoknot module. The sensitivity of group 1 is 87.5% and specificity is 97.06%. For group 2, the sensitivity is 80.52% and specificity is 100%. In addition, the sensitivity is 64.71% and 56.34%, and specificity is 100% and 98.85% for groups 3 and 4, respectively. Thus, this pseudoknot module was required to improve the accuracy of IRES prediction. The VIPS contains RNA pseudoknot prediction module and four individual IRES group alignment functions in a IBM workstation with 2 CPU containing 8 cores on board.

With pseudoknot module, the VIPS significantly increases the sensitivity and accuracy of the prediction for IRES group 1 and 4. For those two groups, the sensitivity and accuracy were enhanced from 87.50% to 100.00% and 92.28% to 98.53% in group 1, and 56.34% to 62.44% and 77.60% to 80.41% in group 4, respectively (Table 1 and 2). The sensitivity and accuracy were also enhanced from 80.52% to 81.59% and 90.26% to 90.80% in group 2. Unfortunately, pseudoknot module does not improve the sensitivity and accuracy for IRES group 3 structures. RNA pseudoknot structure is found in RNA catalysts,folded RNA, ribosome and telomerase. Current evidences showed that pseudoknots act a key structural role in bringing distant regions of single-stranded RNA together to form core helices that were composed with Watson-Crick base pairs [21]. Pseudoknot structures also regulate IRESs, because pseudoknots have been demonstrated to stimulate the efficiency of translational recoding events that include redefined stop codon and ribosomal frameshifting [22]. In addition, pseudoknot containing transfer-messenger RNA (tmRNA) can rescue stalled ribosomes that reached the 3′ end of an mRNA lacking a termination codon during translation elongation [23]. In viruses, pseudoknots have been identified in a number of IRESs and their function has been proven in the flavivirus HCV and the dicistrovirus cricket paralysis virus (CrPV) [3,24]. And, HCV IRES domains function synergistically to locate the AUG sequence into the ribosomal peptidyl (P) site that might couple the movement of the pseudoknot with HCV IRES domain 3d. With pseudoknot, false positive values of VIPS prediction are 2.94%, 0.00%, 0.00%, and 1.63%, and false negative values are 0.00%, 18.41%, 35.29%, and 37.56% both for IRES group 1, 2, 3 and 4, respectively.

The cellular IRESs of IRES database was also analyzed by VIPS, while those IRES structures are confirmed by Rfam database with experimental evidence. The accuracy of cellular IRESs prediction is lower than viral IRESs. The results of VIPS analyzed from UTR database, positive group may contain 39 genes related to different catalogs which might have potential IRES elements. According to COG database [25], those genes containing potential IRES elements can be classified into 18 catalogs. They are 1) translation, ribosomal structure (J, 4.65%); 2) transcription (K, 6.98%); 3) DNA replication, recombination and repair (L, 2.33%); 4) posttranslational modification, protein turnover, chaperones (O, 2.33%); 5) RNA processing and modification (A, 2.33%); 6) Nuclear structure (Y, 2.33%); 7) Extracellular structures (W, 2.33%); 8) Intracellular trafficking, secretion, and vesicular transport (U, 4.65%); 9) inorganic ion transport and metabolism (P, 6.98%); 10) signal transduction mechanisms (T, 16.28%); 11) energy production and conversion (C, 2.33%); 12) carbohydrate transport and metabolism (G, 4.65%); 13) Amino acid transport and metabolism (E, 2.33%); 14) nucleotide transport and metabolism (F, 4.65%); 15) coenzyme metabolism (H, 2.33%); 16) lipid metabolism (I, 4.65%); 17) secondary metabolites biosynthesis, transport and catabolism (Q, 2.33%) and 18) Function unknown (S, 9.3%). Most of the candidate genes are classified into Signal transduction mechanisms [T] and General function prediction only [R] catalogs that are 32.56% of the total candidates. However, more experimental evidences are necessary to prove the function of predicted IRES elements and the relationship between gene expression by IRES and gene catalogs. 

In RNA structure prediction, Rfam provides pattern searching program and web service which was developed by Sanger Institute [16]. Rfam adopts covariance models to estimate consensus primary sequences of non-coding RNA families, thus, Rfam provides information not focus on IRESs. In contrast, VIPS was more specific for IRES study with combination of four well-defined viral RNA models. Thus, VIPS can predict IRESs by structure comparison including pseudoknot which contains neighboring regions for structure prediction to avoid short consensus primary sequence problems that are approached differently by Rfam.

Based on results the obtained from VIPS, Bat coronavirus (NC_010436) and Human enterovirus (NC_013114) are the major members of positive group in group 4. However, positive group may contain other viruses which might have potential IRES elements. For example, Human rhinovirus C (NC_009996) has high R value (1.74) in 423-626 nucleotides. The pseudoknot function will select more candidate IRES elements for group 4, such as Porcine enterovirus B (data not shown). For group 3, Foot-and-mouth disease virus (NC_004915) and Human cosavirus (NC_012802) are the major families of positive group with pseudoknot function. Without pseudoknot prediction, some of the virus families might lose in the current criteria of VIPS. HCV and Hepatitis GB virus B (NC_001655) occupy major percentage in the positive group of VIPS for IRES group 2. Another ssRNA positive strand virus, Dengue virus (NC_001477), has been discovered as potential IRES element with pseudoknots and has been proven by mutagenesis experiments [26]. Without pseudoknot structure, the sensitivity of VIPS is reduced for IRES group 2 due to HCV structure containing pseudoknot. For IRES group 1, Himetobi P virus (NC_003782) showed the highest percentage in the positive group by VIPS (1.93 with pseudoknot score). Moreover, *Diaporthe ambigua* RNA virus 1 (NC_001278), 3947-4111 nucleotides, has potential IRES element (R value is 1.60, without pseudoknot). In group 1 prediction, there is no significant difference with pseudoknot function or not. Recent researches suggest that the Dicistroviridae family might have intergenic IRES from bioinformatic evidence [27] which are matched our predictions. Our results demonstrate that VIPS does not only to predict RNA secondary structures, but also locates the IRES elements in the viral genome. 

In VIPS, pseudoknot prediction was implemented as a criterion because many IRES elements contain pseudoknot structures such as HCV IRES element [12]. However, pseudoknot parameter indicates stable pseudoknot structure or not and then is easy to locate short sub-structure. Therefore, pseudoknot parameter with an R-value prevents overestimation of the predicted IRES elements that can also be revealed as false positive results. After evaluation of pseudoknot parameter by four IRES standard elements of VIPS, pseudoknot parameters can cover known IRES structures and also avoid the disadvantages of minimum free energy method (data not showed). However, to improve sensitivity and specificity of cellular IRES elements in VIPS, new algorithms can be implemented to simulate real relationships and interactions between 40s rRNA and IRES elements in next version of our prediction system. The new bioinformatic tool plays a major role in creating databases and finding eukaryotic functional elements such as IRES, iron-responsive elements, splicing regulatory elements.. etc [28]. Therefore, VIPS will be a useful internet resource for IRES elements location before experimental studies. Moreover, it can facilitate the scientific community not only to study IRES using VIPS, but also as means of communication by providing some feedbacks.

## Conclusions

Computational prediction of IRES element is difficult to find the appropriated software. We have designed a viral IRES prediction system (VIPS) to perform the four groups of IRES predictions. To generate more specific prediction results, VIPS integrated RNA secondary structure prediction program, comparison software and pseudoknot program to increase the accuracy rate for IRES elements prediction. VIPS can facilitate users to quickly identify candidate IRES structures from their target sequences. The ability of VIPS to perform single sequence input and the availability of online service renders a high flexibility in its application.

## Supporting Information

Figure S1 **The output format of each program in VIPS.** The input and out format of RNAL fold, RNA Align and pknoUsRG were showed.
(TIF)

Methods S1 **Program perl/R script: Start_analyze.pl, UTR2SQ.pl, utr_dp.pl, B2RA.pl, B2CT.pl, run_ pknotsRG.pl, DIST.R and sort.** **R**. A perl source code represents the program to transfer the sequences into VIPS and re-format the input/output of RNAL fold, RNA Align and pknotsRG. And, R source code represents the program to analyze all alignment scores, calculate the score distribution and transform the output data from DIST.R into a table format which can be read by Microsoft^®^ Word^®^ program.
(DOC)

Table S1 **All positive records of four IRES groups searched by VIPS.** A table that lists all of the verified IRES elements of our IRES groups used for VIPS study.
(XLS)

Table S2 **The cross comparison of the performance of four IRES group with each other.** The standard IRES elements were examined and compared with different IRES groups by VIPS.
(XLS)

Table S3 **The Zinc finger genes from those IRES group 1 and 4 candidates.** Using VIPS, the listed Zinc finger genes from IRES groups 1 and 4 candidates were searched in UTR database.
(XLS)

Table S4 **The potential IRES elements from selected 500 viral genomes analyzed by VIPS.** The random selected 500 viral genome sequences were analyzed using VIPS. The excel table showed the pseudoknot structure and R scores of each IRES group around candidate regions of viral genomes after prediction by VIPS.
(XLS)
