# Supplementary figures and images for: Viral IRES Prediction System - a Web Server for Prediction of the IRES Secondary Structure In Silico
Source: PLoS One. 2013 Nov 5;8(11):e79288. doi: 10.1371/journal.pone.0079288 (PMC3818432; doi:10.1371/journal.pone.0079288)

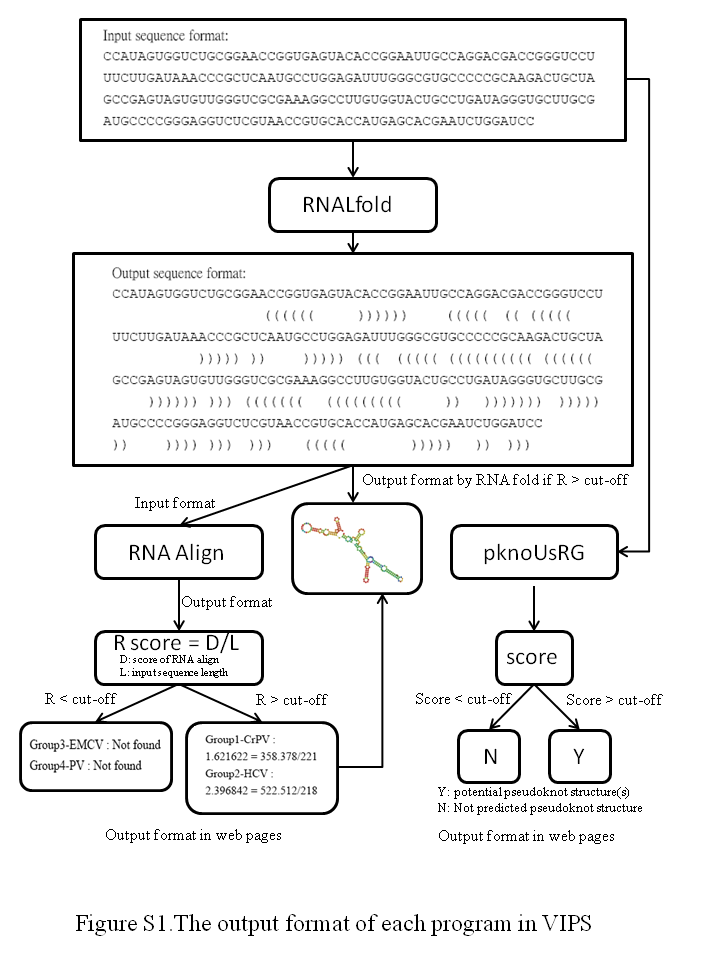

Supplement: Figure S1 — The output format of each program in VIPS. The input and out format of RNAL fold, RNA Align and pknoUsRG were showed. (TIF) [file pone.0079288.s001.tif]
