# Supplementary material for: Viral IRES Prediction System - a Web Server for Prediction of the IRES Secondary Structure In Silico
Source: PLoS One. 2013 Nov 5;8(11):e79288. doi: 10.1371/journal.pone.0079288 (PMC3818432; doi:10.1371/journal.pone.0079288)
Supplement: Methods S1 — Program perl/R script: Start_analyze.pl, UTR2SQ.pl, utr_dp.pl, B2RA.pl, B2CT.pl, run_ pknotsRG.pl, DIST.R and sort. R. A perl source code represents the program to transfer the sequences into VIPS and re-format the input/output of RNAL fold, RNA Align and pknotsRG. And, R source code represents the program to analyze all alignment scores, calculate the score distribution and transform the output data from DIST.R into a table format which can be read by Microsoft® Word® program. (DOC) [file pone.0079288.s002.doc]

1. UTR2SQ.pl

#!/usr/bin/perl

use strict;

use warnings;

use File::Basename;

use lib dirname __FILE__;

use CCHLOG;

if ($#ARGV >= 0) { # 0 means 1 argument

unless (-e $ARGV[0]) {

print "Config file \'$ARGV[0]\' not found! Abort! $!\n";

exit;

}

} else {

print 'Usage:', "\n";

print ' UTR2SQ.pl ConfigFileName [Start [End]]', "\n";

exit;

}

open (CFGFH, $ARGV[0]) or die "Can't open config file \'$ARGV[0]\'! $!\n";

chomp (my $datfilename = <CFGFH>);

chomp (my $outputpath = <CFGFH>);

mkdir ($outputpath, 0777) or die "Cannot mkdir $outputpath: $!";

my $total_file = "./$outputpath/total.txt";

my $BTF_file = "./$outputpath/BTF.err";

chomp (my $outputlogfilename = <CFGFH>);

my $logfilename = "./$outputpath/$outputlogfilename";

chomp (my $param = <CFGFH>);

chomp (my $call_utr_dp = <CFGFH>);

my $dpcfgfile;

if ($call_utr_dp eq 'y') {

chomp ($dpcfgfile = <CFGFH>);

}

close (CFGFH);

# Start sequence number (default = 1)

my $start_num = 1;

if ($ARGV[1]) {

$start_num = $ARGV[1];

}

# End sequence number (default = not define = end of file)

my $end_num;

if ($ARGV[2]) {

$end_num = $ARGV[2];

}

open (CFGFH, $ARGV[0]);

my @totalcfg = <CFGFH>;

close (CFGFH);

vislog ($logfilename, "@ARGV\n@totalcfg\n");

#-------------------------------------------------------------------

# Start

open (FH, $datfilename) || die "Could not open $datfilename!";

vislog ($logfilename, localtime()." UTR2SQ.pl\n");

my $acc_num;

my $seq_count = 0;

my $str_count = 0;

while (my $item = <FH>) {

my @items = split ' ', $item;

if ($items[0] eq 'ID') {

# new sequence

$seq_count++;

if (($end_num) && ($seq_count > $end_num)){

last;

}

} elsif ($items[0] eq 'AC') {

if ($seq_count >= $start_num) {

$acc_num = $items[1];

chomp $acc_num;

chop $acc_num; # drop ';'

repvislog ($logfilename,

sprintf ("\n%10d AC: %s\n", $seq_count, $acc_num), '.');

}

} elsif ($items[0] eq 'SQ') {

if ($seq_count >= $start_num) {

invislog ($logfilename,

sprintf (" SQ: length: %d\n", $items[2]));

# Get one sequence form input file.

my $seq;

for (my $i = 0; $i < $items[2]/60; $i++) {

my $seq_line = <FH>;

my @seq_line_items = split ' ', $seq_line;

pop @seq_line_items; # drop last item (numbering)

my $seq_one_line = join '', @seq_line_items;

$seq .= $seq_one_line;

}

# Open an temp file and print the sequence into temp file

my $tempfile = 'temp';

opcfile ('>'.$tempfile, ">$acc_num\n$seq");

# Structure prediction of the sequence in temp file

system "RNALfold $param < temp > $acc_num.txt";

unlink $tempfile;

my $temp_count = count ("$acc_num.txt");

# Test if structure prediction was failed for this sequence..

if ($temp_count > 0) {

append_file ($total_file, $acc_num.'.txt', 'AC '.$acc_num);

append_string ($total_file, "END\n");

}

unlink $acc_num.'.txt';

$str_count += $temp_count;

} # if ($seq_count >= $start_num)

} # elsif ($items[0] eq 'SQ')

} # while (my $item = <FH>)

close FH;

my $real_seq_count = $seq_count - $start_num;

vislog ($logfilename, "\nSequence count: $real_seq_count\n");

vislog ($logfilename, "Structure count: $str_count\n");

if ($call_utr_dp eq 'y') {

exec "perl ../../bin/utr_dp.pl $dpcfgfile";

}

exit;

# --------------------------------------------------------

# Program-wide subroutine

sub count {

my $file = shift;

open (TEMPFH, $file) || die "Could not open $file to count!";

my $found_structures = 0;

my $line = <TEMPFH>; # drop first line

my $line_count = 1;

while (<TEMPFH>) {

$line_count++;

chomp;

my @line_items = split; # must be split

if ($line_items[0] =~ m/\(/) { # search for '('

$found_structures++;

} elsif ($line_items[0] =~ m/[agct]/i) {

# end if we meet ATGC... etc.

last;

} else {

invislog ($logfilename,

"Line $line_count contain no structure!\n");

}

}

close TEMPFH;

if ($line_count == 1) {

invislog ($logfilename,

"BTF in f3\n");

invislog ($BTF_file, "$acc_num\n");

unlink $acc_num.'.txt';

}

return $found_structures;

}

# --------------------------------------------------------

# System wide subroutine

sub opcfile {

my $tempfile = shift;

my $print_string = shift;

open (TEMPFH, $tempfile) or

die "opcfile(): Could not open $tempfile!";

print TEMPFH $print_string;

close TEMPFH;

}

2. utr_dp.pl

#!/usr/bin/perl -w

# --------------------------------------------------------

use strict;

use warnings;

#use File::Copy;

use File::Basename;

use lib dirname __FILE__;

use CCHLOG;

use B2RA;

print @ARGV,"\n", $#ARGV, "\n";

if ($#ARGV >= 0) { # 0 means 1 argument

unless (-e $ARGV[0]) {

print "Config file \'$ARGV[0]\' not found! Abort! $!\n";

exit;

}

} else {

print 'Usage:', "\n";

print ' utr_dp.pl ConfigFileName [Start [End]]', "\n";

exit;

}

open (CFGFH, $ARGV[0]) or die "Can't open config file \'$ARGV[0]\'! $!\n";

my $outputpath = <CFGFH>; chomp $outputpath;

my $templatefile = <CFGFH>; chomp $templatefile;

unless (-e $templatefile) {

qlog ("We need template file: \'$templatefile\'!");

die;

}

my $call_next = <CFGFH>; chomp $call_next;

close (CFGFH);

# --------------------------------------------------------

my $starttime = time;

my $log_file = "./$outputpath/utr_dp.log";

my $total_ali = "./$outputpath/total.ali";

my $total_alilog = "./$outputpath/total.ali.log";

my $total_file = "./$outputpath/total.txt";

unlink $log_file;

unlink $total_ali;

unlink $total_alilog;

my $score_file = "./$outputpath/score.csv";

unlink $score_file;

invislog ($score_file, "AC, START_POS, STRU_LEN, DIST, ALI_LEN, RATIO\n");

my $start_num = 1;

if ($ARGV[1]) {

$start_num = $ARGV[1];

}

my $end_num; # undefine $end_num means reach the end!

if ($ARGV[2]) {

$end_num = $ARGV[2];

}

# --------------------------------------------------------

my $OS = check_OS();

qlog (localtime()." utr_dp $OS");

# --------------------------------------------------------

open (CFGFH, $ARGV[0]);

my @totalcfg = <CFGFH>;

close (CFGFH);

qlog ("@ARGV\n@totalcfg\n");

# --------------------------------------------------------

open (TOTAL, $total_file) || die "Can't open total.txt!!";

my $seq_count = 0;

my $stru_count = 0;

while (<TOTAL>) {

my $AC;

my $name;

my @lineitems = split ' ', $_;

# Every sequence start with AC and end with END

# Here we cut total.txt into pieces of sequence

if ($lineitems[0] eq 'AC') {

$seq_count++;

if (($end_num) && ($seq_count > $end_num)){

last;

}

$AC = $lineitems[1];

my $next_line;

my $write_sequence;

do {

$next_line = <TOTAL>;

unless ($next_line =~ m/^END/) {

$write_sequence .= $next_line;

}

} until ($next_line =~ m/^END/);

unless ($seq_count >= $start_num) {

next;

}

qlog ($AC.' '.localtime().' '.$seq_count);

# write file

$name = $AC.'.txt';

open (TOTALTEMP, '>'.$name) || die "Can't open $name!!: $!";

print TOTALTEMP $write_sequence;

close TOTALTEMP;

} else {

qlog ("Strange Format?");

next;

}

#---------------------------------------------------------------

# Calculate valid structures in sequence.

# Prepare structures.

open (TEMP, $name) || die "Can't open $name?";

<TEMP>; #drop first line

my @structs;

my $code;

my $found_structures = 0;

my $line_count = 1; # because we drop first line.

while (<TEMP>) {

chomp;

$line_count++;

my @line_items = split; # must be split

if ($line_items[0] =~ m/\(/) { # search for '('

#my @out = split (//,$_);

#print '['.$line_items[0],"]\n";

$found_structures++;

push @structs, $_;

} elsif ($line_items[0] =~ m/[agct]/i) {

# end if we meet ATGC... etc.

$code = $_;

last;

} else {

print "Line $line_count contain no structures!\n";

}

}

close (TEMP);

unlink $name;

qlog ($found_structures.' structure(s).');

#---------------------------------------------------------------

# we can do b2ra and run rna_align now

if (($code) && ($found_structures > 0)) {

my $count = 0;

foreach my $item (@structs) {

# my @temp = split (" ", join ("", @{$item})); # re-split by " "

# my @br = split("",$temp[0]);

# my $len = $#br + 1;

# my $code_start = $temp[$#temp]-1;

# my $code_end = $code_start + $#br;

# my @tempcodes = @codes[$code_start..$code_end];

$count++;

@_ = split ' ', $item;

my $br = $_[0];

my $len = length($br);

my $code_start = $_[$#_]-1;

my $tempcode = substr ($code, $code_start, $len);

br2ra ($br, $tempcode, 'temp.ra');

#B2RA::br2ct (\@br, \@tempcodes, 'temp.ct');

$stru_count++;

#---------------------------------------------------------------

# Call DP

qlog ('DP POS:'.($code_start+1).' '.$count.'/'.$found_structures.' '.$seq_count.' '.$stru_count.' ['.localtime().']');

my $dp_command =

"dp $templatefile temp.ra original.param temp.ali > temp.ali.log";

if ($OS eq 'UNIX') {

$dp_command = "./".$dp_command;

}

my $ret_val = system $dp_command;

getrascore ('temp.ali.log', $AC, $code_start+1, $len);

append_ali ($AC, $code_start+1, $len);

if ($ret_val != 0) {

qlog ('DP failed and crashed at '.localtime());

# Although DP crashed, we still try

# to finish other calculations.

}

#---------------------------------------------------------------

#append_ra_ct ($AC, $temp[$#temp], $#br+1); # AC, Start_Pos, Length

unlink 'temp.ra'; #unlink 'temp.ct';

} # foreach my $item (@structs)

} # if ((@codes) && ($found_structures > 0))

} # while (<TOTAL>)

close (TOTAL);

my $endtime = time;

my $diff_time = $endtime - $starttime;

print $diff_time, "\n";

# Use gzip and tar to pack results

pack_dir ($outputpath);

if ($call_next eq 'y') {

if (-e "next.pl") {

rename "next.pl", "next.pl.done";

exec "perl ./next.pl.done";

}

}

exit;

# ========================================================

# --------------------------------------------------------

# Program-wide subroutine

sub qlog {

my $log_string = shift;

vislog ($log_file ,$log_string."\n");

}

#sub append_ra_ct {

# my $name = shift;

# my $start_pos = shift;

# my $len = shift;

#

# my $sep_string = 'AC: '.$name.' POS: '.$start_pos.' LEN: '.$len;

#

# append_file ($total_ra_file, 'temp.ra', $sep_string);

# append_file ($total_ct_file, 'temp.ct', $sep_string);

#}

sub append_ali {

my $name = shift;

my $start_pos = shift;

my $len = shift;

my $sep_string = 'AC: '.$name.' POS: '.$start_pos.' LEN: '.$len;

append_file ($total_ali, 'temp.ali', $sep_string);

append_file ($total_alilog, 'temp.ali.log', $sep_string);

}

# getrascore --------------------------------------------------------

sub getrascore {

my $ra_log_file = shift;

my $AC = shift;

my $start = shift;

my $slen = shift;

open (RALOGFH, $ra_log_file) || die "Couldn't open $ra_log_file!";

my $prev_line;

my $last_line;

while (my $line = <RALOGFH>) {

$prev_line = $last_line;

$last_line = $line;

}

close RALOGFH;

my @last_line_items = split '=', $last_line;

if ($last_line_items[0] eq 'length') {

chop $prev_line;

my $len = $last_line_items[1]/2;

open (SCOREFH, '>>'.$score_file) || die "Couldn't open $score_file!";

printf "$AC, %8d, score: %d, length: %d, l/s: %8f\n",

$start, $prev_line, $len, $len/$prev_line;

printf SCOREFH "$AC, %8d, %8d, %8f, %8d, %8f\n",

$start, $slen, $prev_line, $len, $len/$prev_line; #prev_line=score,(distance)

close SCOREFH;

} else {

print "Failed! $!";

}

}

sub pack_dir {

my $dir = shift;

if (-e "./$dir/total.txt") {

system "gzip ./$dir/total.txt";

}

system "gzip ./$dir/total.ali";

system "gzip ./$dir/total.ali.log";

system "tar -cvf $dir.tar $dir";

system "gzip $dir.tar";

}

# --------------------------------------------------------

# System wide subroutine

sub check_OS {

my $OS = '';

unless ($OS) {

unless ($OS = $^O) {

require Config;

$OS = $Config::Config{'osname'};

}

}

if ($OS =~ /^MSWin/i) {

$OS = 'WINDOWS';

} elsif ($OS =~ /^VMS/i) {

$OS = 'VMS';

} elsif ($OS =~ /^dos/i) {

$OS = 'DOS';

} elsif ($OS =~ /^MacOS/i) {

$OS = 'MACINTOSH';

} elsif ($OS =~ /^os2/i) {

$OS = 'OS2';

} elsif ($OS =~ /^epoc/i) {

$OS = 'EPOC';

} elsif ($OS =~ /^cygwin/i) {

$OS = 'CYGWIN';

} else {

$OS = 'UNIX';

}

return $OS;

}

3. B2RA.pl

#!/usr/bin/perl -w

use strict;

use warnings;

use File::Basename;

use lib dirname __FILE__;

use B2RA;

my $name = $ARGV[0];

my $output_name = basename ($name).".ra";

if ($ARGV[1]) {

$output_name = $ARGV[1];

}

open (TEMP, $name) || die "Can't open $name?";

<TEMP>; #drop first line

my @structs;

my $code;

my $found_structures = 0;

my $line_count = 1; # because we drop first line.

while (<TEMP>) {

chomp;

$line_count++;

my @line_items = split; # must be split

if ($line_items[0] =~ m/\(/) { # search for '('

$found_structures++;

push @structs, $_;

} elsif ($line_items[0] =~ m/[agct]/i) {

# end if we meet ATGC... etc.

$code = $_;

last;

} else {

print "Line $line_count contain no structures!\n";

}

}

close (TEMP);

if (($code) && ($found_structures > 0)) {

my $count = 0;

foreach my $item (@structs) {

$count++;

@_ = split ' ', $item;

my $br = $_[0];

my $len = length($br);

my $code_start = $_[$#_]-1;

my $tempcode = substr ($code, $code_start, $len);

br2ra ($br, $tempcode, $output_name);

}

}

4. B2CT.pl

#!/usr/bin/perl -w

use strict;

use warnings;

use File::Basename;

use lib dirname __FILE__;

use B2CT;

my $name = $ARGV[0];

my $output_name = basename ($name).".ct";

if ($ARGV[1]) {

$output_name = $ARGV[1];

}

open (TEMP, $name) || die "Can't open $name?";

<TEMP>; #drop first line

my @structs;

my $code;

my $found_structures = 0;

my $line_count = 1; # because we drop first line.

while (<TEMP>) {

chomp;

$line_count++;

my @line_items = split; # must be split

if ($line_items[0] =~ m/\(/) { # search for '('

$found_structures++;

push @structs, $_;

} elsif ($line_items[0] =~ m/[agct]/i) {

# end if we meet ATGC... etc.

$code = $_;

last;

} else {

print "Line $line_count contain no structures!\n";

}

}

close (TEMP);

if (($code) && ($found_structures > 0)) {

my $count = 0;

foreach my $item (@structs) {

$count++;

@_ = split ' ', $item;

my $br = $_[0];

my $len = length($br);

my $code_start = $_[$#_]-1;

my $tempcode = substr ($code, $code_start, $len);

br2ct ($br, $tempcode, $output_name);

}

}

5. start_analyze.pl

#! /usr/bin/perl

use strict;

my %info = &loadINFOfile('info.txt'); # Load input item

my $id = $info{'ID'};

my $seq = $info{'Sequence'};

my @selectGroup;

my %threshold;

foreach my $group('CrPV','HCV','EMCV','PV'){

my $groupkey = "$group".'group';

my $thresholdkey = "$group".'threshold';

if ($info{$groupkey} eq 'on'){

push( @selectGroup,$group);

$threshold{$group} = $info{$thresholdkey};

}

}

&firstcheck;

###--Parameter--###

open (CFG,'example.250.2s.cfg') || die $!;

my @cfg = <CFG>;

close CFG;

open (DPCFG,'example.dp.cfg') || die $!;

my @dpcfg = <DPCFG>;

close DPCFG;

chomp $cfg[3];

chomp $dpcfg[1];

chomp $dpcfg[2];

$cfg[3] =~ m/-L\s/o;

my $foldlen = $'; #example.250.2s.cfg line4. RNALfold fold length

my $threads_num = $dpcfg[2]; #example.dp.cfg line4. threads number

if ($threads_num <= 0){

#print "EXIT: The threads number <= 0 !!\n";

exit;

}else {

#print "The threads number is $threads_num\n\n";

}

my $pstcond; #PseudoknotCondition = 'Y' || 'N'

if ($info{'PseudoknotCondition'} eq 'on'){

$pstcond = 'Y';

}elsif ($info{'PseudoknotCondition'} eq 'off'){

$pstcond = 'N';

}

if (!$pstcond){

$pstcond = 'N';

}

###-- Start analysis --###

if (!-e "$id"){

mkdir ("$id",0777);

}else{

mkdir ("$id",0777) if (!-d "$id");

}

# create RNA_seq file

open (RNA,'>RNA_seq') || die $!;

print RNA "\>$id\n$seq";

close RNA;

my $outputfile = 'results/results';

foreach my $iresgroup(@selectGroup){

### create template.cfg file ###

open (TM,'>template.cfg') || die $!;

print TM './template/',"$iresgroup",'_template.ra';

close TM;

###--System order--###

my $call_UTR2SQ = "perl ../../bin/UTR2SQ.pl example.250.2s.cfg 1 1";

my $call_run_pknotsRG = "perl ../../bin/run_pknotsRG.pl $outputfile $threshold{$iresgroup} $threads_num $iresgroup"; #result output to "$outputfile"

###--Call the analysis program--###

#print "\ncalculation 2D structural similarity...\n";##

system "$call_UTR2SQ";

&sortResults('results/score.csv',"$threshold{$iresgroup}",'results/results','RNA_seq');

if ($pstcond eq 'Y'){

#print "\ncalculation pseudoknot structure...\n";##

system "$call_run_pknotsRG";

}

###--Delete file & move results--###

system "mv results $id/Compare_to_$iresgroup";

unlink 'results.tar.gz';

}

###--Delete file--###

unlink 'RNA_seq';

unlink 'template.cfg';

system "mv info.txt $id/info.txt";

### check folder name && move results folder to ../results/ ###

my $optfoldername = $id;

my @existsfolder = glob "../results/$optfoldername*";

@existsfolder = grep {-d "$_" && $_ =~ m/$optfoldername\-*\d*/} @existsfolder;

if (@existsfolder){

my $idx = 0;

foreach my $folder(@existsfolder){

if ($folder =~ m/$optfoldername\-(\d+)/){

$idx = $1 if ($1 > $idx);

}

}

$idx++;

$optfoldername .= "\-$idx";

}

system "mv $id ../results/$optfoldername";

###--Sendmail--###

system "perl sendmail.pl ../results/$optfoldername";

#print "\nFinish!!\n\n";##

exit;

############################################

sub loadINFOfile{

my $filename = shift;

open (FH,"$filename") || die $!;

my %info;

while (my $line = <FH>){

chomp $line;

$line =~ s/\s//g;

next if ($line !~ m/\w|\d/g);

my ($item,$content) = split '=',$line;

if ($item eq 'Sequence'){

while (my $seq = <FH>){

chomp $seq;

$seq =~ s/\s//g;

last if ($seq !~ m/\w|\d/g);

$content .= $seq;

}

}

$info{$item} = $content;

}

close FH;

return %info;

}

sub firstcheck{

if (-e "../results" && !-d "../results"){

print "The ../results is not a folder!! Create the folder\n";

mkdir ("../results/",0777);

}elsif (!-e "../results"){

print "The ../results/ folder is not exists!! Create the folder\n";

mkdir ("../results/",0777);

}

foreach my $templatefile('CrPV_template.ra','HCV_template.ra','EMCV_template.ra','PV_template.ra'){

#'CrPV_ires.fa','HCV_ires.fa','EMCV_ires.fa','PV_ires.fa'

if (!-e "template/$templatefile"){

print "EXIT: template/$templatefile file is not exists!!\n";

exit;

}

}

### reseting ###

foreach my $checkfile('results','results.tar.gz'){

if (-e "$checkfile"){

if (-d "$checkfile"){

system "rm -r $checkfile";

}else{

unlink $checkfile;

}

}

}

my @ORFfile = glob "ORF*";

foreach my $ORFfile(@ORFfile){

unlink $ORFfile;

}

### chechk other file ###

}

sub delfile{

unlink "ORF\d" if (-e "ORF\d");

}

sub sortResults{

my $scorefile = shift;

my $threshold = shift;

my $outputfile = shift;

my $seqfile = shift;

my $seq;

open (SQ,"$seqfile") || die $!;

while (my $seqline = <SQ>){

chomp $seqline;

if ($seqline =~ m/^\>/){

while ($seqline = <SQ>){

chomp $seqline;

last if ($seqline =~ m/^\>/);

$seq .= $seqline;

}

}

last if ($seqline =~ m/^\>/);

}

close SQ;

$seq =~ s/\s//g;

my $output;

my $n = 0;

open (FH,"$scorefile") || die $!;

my $title = <FH>;

while (my $line = <FH>){

chomp $line;

$line =~ s/\s//g;

my ($ac,$start,$len,$dist,$alilen,$ratio) = split ',',$line;

if ($ratio >= $threshold){

my $end = $start + $len - 1;

my $startbs = $start;

if ($startbs >= 1){

$startbs -= 1;

}else {

$startbs = 0;

}

my $fragment = substr ($seq,$startbs,$len);

$output .= "$ac|$start--$end|$ratio\n$fragment\n\n";

$n++;

}

}

close FH;

if ($n == 0){

$output = 'Not find IRES structure';

}

open (OPT,">$outputfile") || die $!;

print OPT "$output";

close OPT;

}

6. run_pknotsRG.pl

#! /usr/bin/perl

use strict;

use threads;

my $datafile = 'results/score.csv';

my $seqfile = 'RNA_seq';

my $outfile = 'ORF';

my $RGtempfile = 'results/RGresults';

my $resultsoptfile = "$ARGV[0]";

my $Rlimit = "$ARGV[1]"; # Ratio limit

my $threads_num = "$ARGV[2]"; # threads number

my $iresgroup = "$ARGV[3]"; # the ires group

my %pstscore = (

'CrPV' => 0.072076,

'HCV' => 0.29,

'EMCV' => 0,

'PV' => 0.018208);

##--Get foldform--##

my $s = 0;

my @threads;

my $thn = 1;

open (FH,"$datafile") || die $! ;

chomp (my $title = <FH>);

while (my $text = <FH>) {

chomp $text;

$text =~ s/\s//g;

my ($ac,$start,$len,$dist,$alilen,$ratio) = split ",",$text;

unless ($ac eq '' | $ac eq 'AC') {

if ($ratio >= ($Rlimit-$pstscore{$iresgroup})){

my $seq = &getRNAseq("$seqfile");

my $end = $start + $len - 1;

my $startbs = $start;

if ($startbs>= 1){

$startbs -= 1;

}else {

$startbs = 0;

}

my $flodseq = substr ($seq,$startbs,$len);

my $foldfrm = "\>$ac\|$start--$end\|$ratio\n$flodseq\n"; #title

##--Output to ORF--##

open (OUT,">$outfile$thn") || die $!;

print OUT "$foldfrm\n";

close (OUT);

$s++;

$threads[$thn] = threads->new(sub{

system "pknotsRG <$outfile$thn >>$RGtempfile";

unlink "$outfile$thn";

});

if ($thn < $threads_num){

$thn++;

}else{

foreach my $t (threads->list()) {

$t->join;

}

$thn = 1;

}

}

}

}

close (FH);

foreach my $t (threads->list) {

$t->join;

}

##--Check results file--##

if ($s >= 1){

&sortRGresults("$RGtempfile","$resultsoptfile",$Rlimit,$pstscore{$iresgroup});

}else{

open (OUT, ">$resultsoptfile") || die $!;

print OUT 'Not find IRES structure';

close (OUT);

}

unlink $RGtempfile;

exit;

#######################################

sub getRNAseq {

my $seq;

my $seqfile = shift;

open (SQ,"$seqfile") || die $!;

while (my $seqline = <SQ>){

chomp $seqline;

if ($seqline =~ m/^\>/){

while ($seqline = <SQ>){

chomp $seqline;

last if ($seqline =~ m/^\>/);

$seq .= $seqline;

}

}

last if ($seqline =~ m/^\>/);

}

close SQ;

$seq =~ s/\s|\d//g;

return $seq;

}

sub sortRGresults{

my $RGfile = shift;

my $optfile = shift;

my $Rlimit = shift;

my $pstscore = shift;

my $match = '\{|\}|\[|\]';

my $output;

my $n = 0;

open (CH,"$RGfile") || die $!;

while (my $line = <CH>){

chomp $line;

if ($line =~ m/^\>(.+)\|(\d+\-\-\d+)\|(.+)/){

chomp (my $seq = <CH>);

chomp (my $fold = <CH>);

my ($ac,$site,$ratio) = ($1,$2,$3);

my $pst;

if ($fold =~ m/$match/o){

$pst = 'Y';

$ratio += $pstscore;

}else{

$pst = 'N';

}

if ($ratio >= $Rlimit){

$output .= "$ac\|$site\|$ratio\|$pst\n$seq\n\n";

$n++;

}

}

}

close CH;

if ($n == 0){

$output = 'Not find IRES structure';

}

open (OUT,">$optfile") || die $!;

print OUT "$output";

close OUT;

}

7. DIST.R

#

test <- read.csv("1-.csv", col.names=c("ac", "startpos", "slen","score", "alen", "ratio"))

attach(test)

# Low level plot

# Ratio

plot.new();

plot.window(xlim=c(1,2.75), ylim=c(0,6))

axis (1);axis (2);box();

lines (density(ratio,bw=0.001))

title (xlab="Ratio", ylab="Count");

rug(ratio)

# DIST (score)

plot.new();

plot.window(xlim=c(90,250), ylim=c(0,0.01))

axis (1);axis (2);box();

lines (density(score,bw=0.5))

title (xlab="Distance score", ylab="Count");

rug(score)

plot (density(score,bw=0.25), xlim=c(90,250))

# ALEN

plot.new();

axis (1);axis (2);box();

plot (density(alen,bw=0.5), xlim=c(200,320))

rug(alen)

# SLEN

plot.new();

axis (1);axis (2);box();

plot (density(slen,bw=0.5), xlim=c(0,250))

rug(slen)

8. sort.R

test <- read.csv("1-.csv", col.names=c("ac", "startpos", "slen","score", "alen", "ratio"))

ratio <- sort (test$ratio, decreasing=TRUE, index.return=TRUE)

out <- data.frame(ac=test$ac[ratio$ix], startpos=test$startpos[ratio$ix], slen=test$slen[ratio$ix], score=test$score[ratio$ix], alen=test$alen[ratio$ix], ratio=test$ratio[ratio$ix])

write.table(out, file = "1-.sorted.csv", sep = ",")
